# Supplementary figures and images for: Limited impact of schistosome infection on Biomphalaria glabrata snail microbiomes
Source: Parasit Vectors. 2026 Feb 26;19:143. doi: 10.1186/s13071-026-07299-z (PMC13041339; doi:10.1186/s13071-026-07299-z)

Kaplan–Meier Survival Curve

Tray    CA    CB    EA    EB

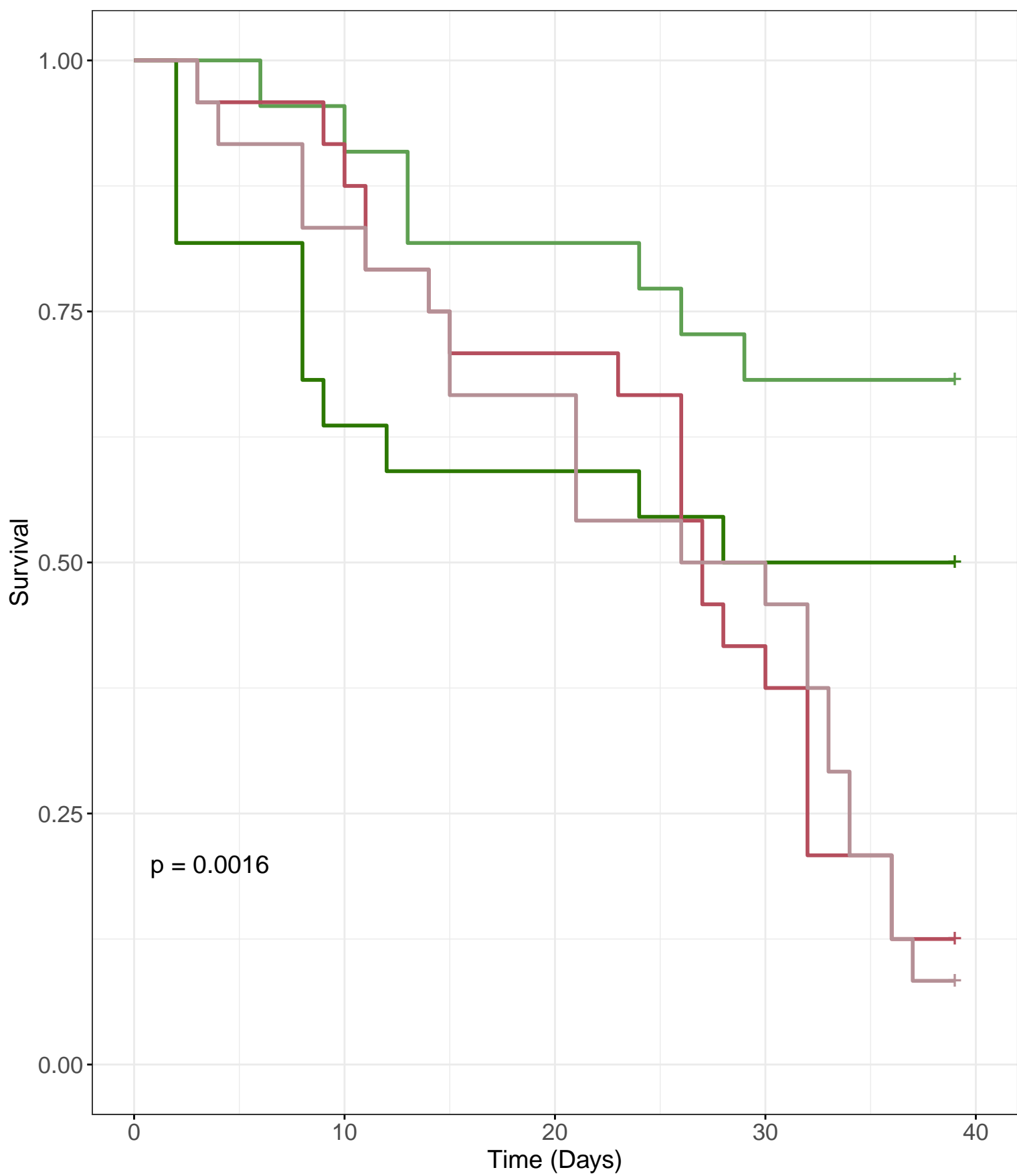

Supplement: Supplementary file 4 — Supplementary material 4: Figure 1. Survival curve. [file 13071_2026_7299_MOESM4_ESM.pdf]

Input Reads

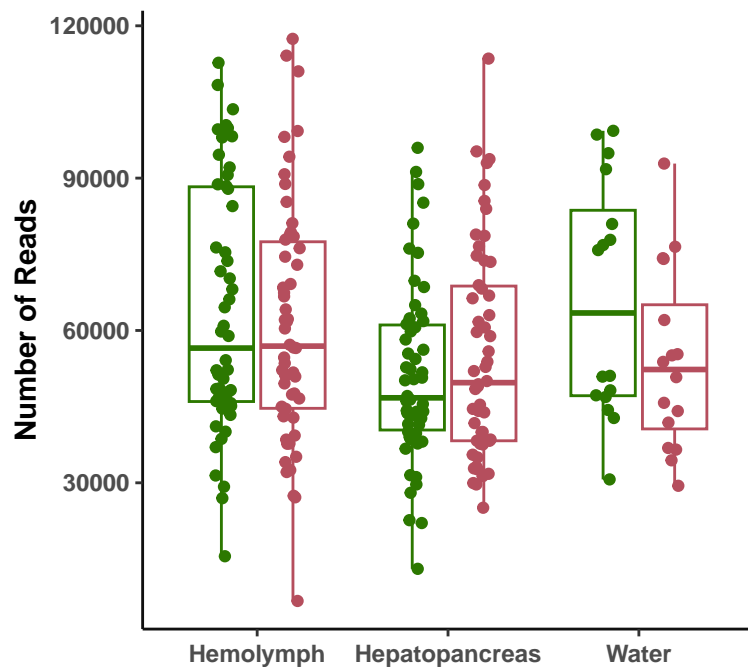

Filtered Reads

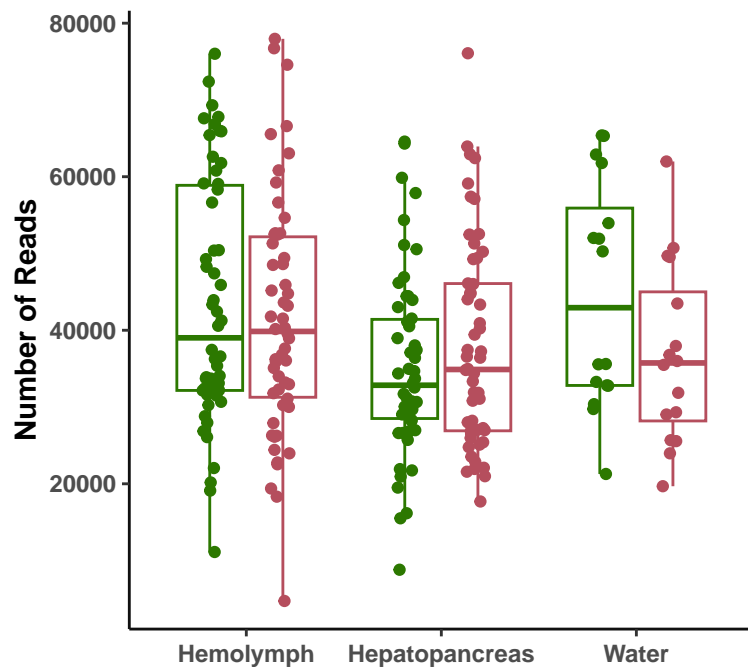

Denoised Reads

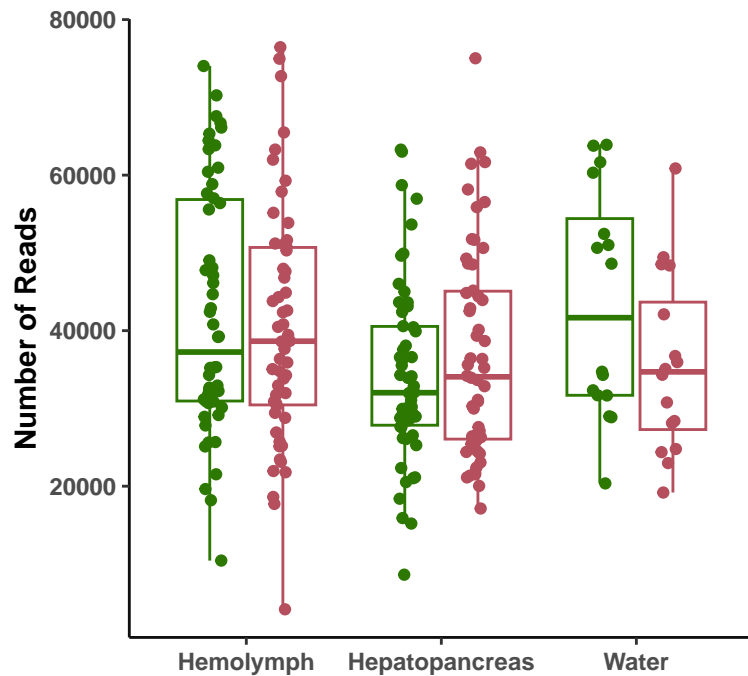

Non-Chimeric Reads

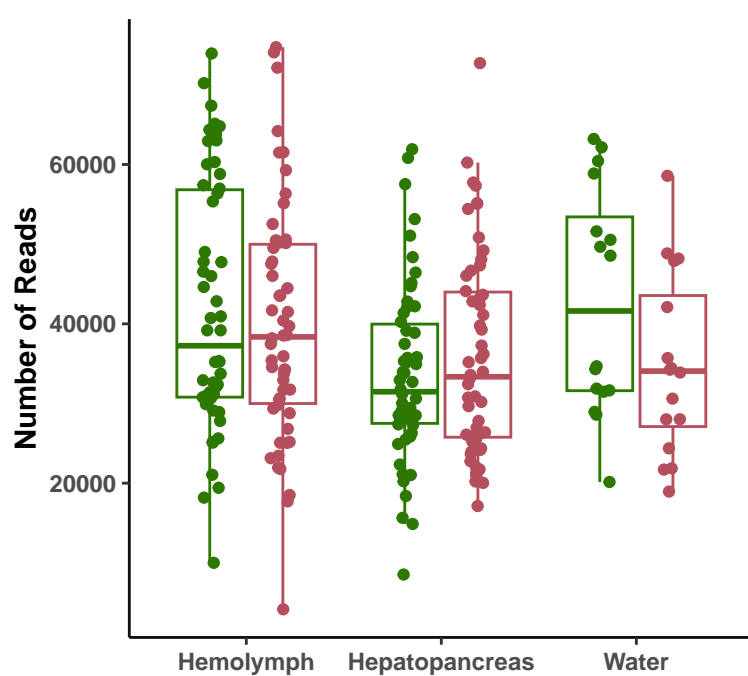

Cohort 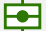 Control 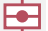 Exposed

Supplement: Supplementary file 5 — Supplementary material 5: Figure 2. Average reads. [file 13071_2026_7299_MOESM5_ESM.pdf]

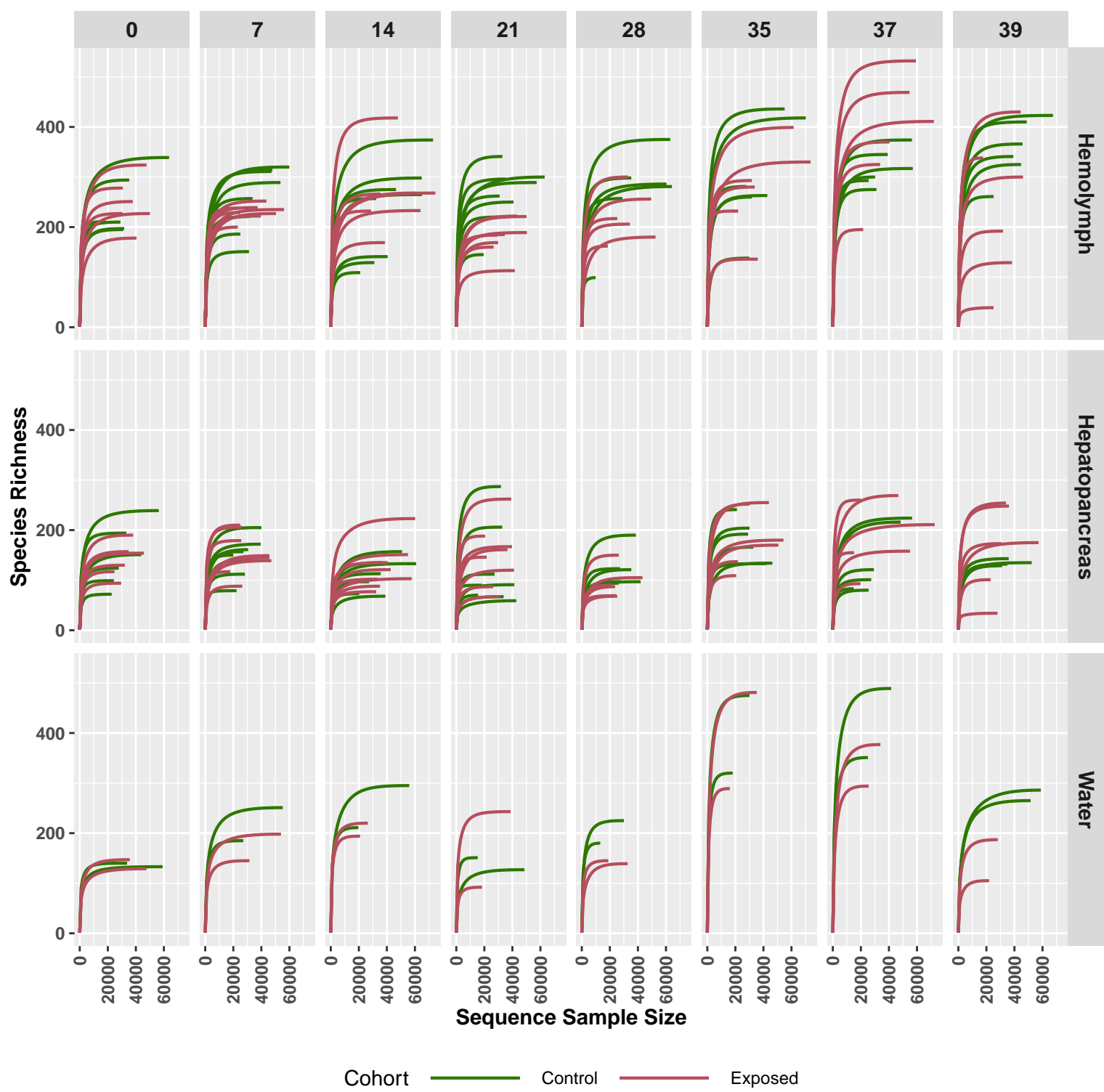

Supplement: Supplementary file 6 — Supplementary material 6: Figure 3. Rarefaction curves. [file 13071_2026_7299_MOESM6_ESM.pdf]

A

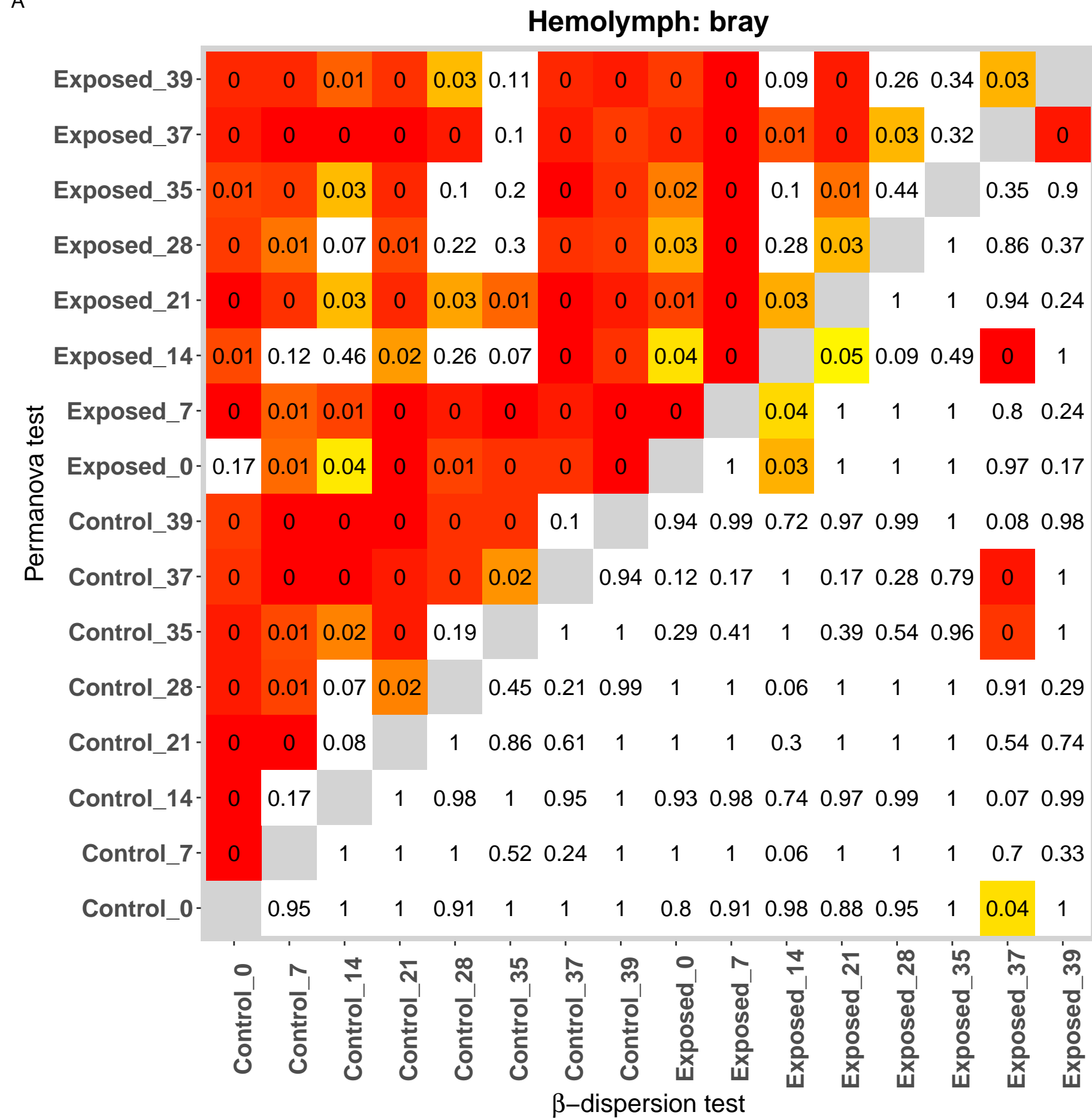

B

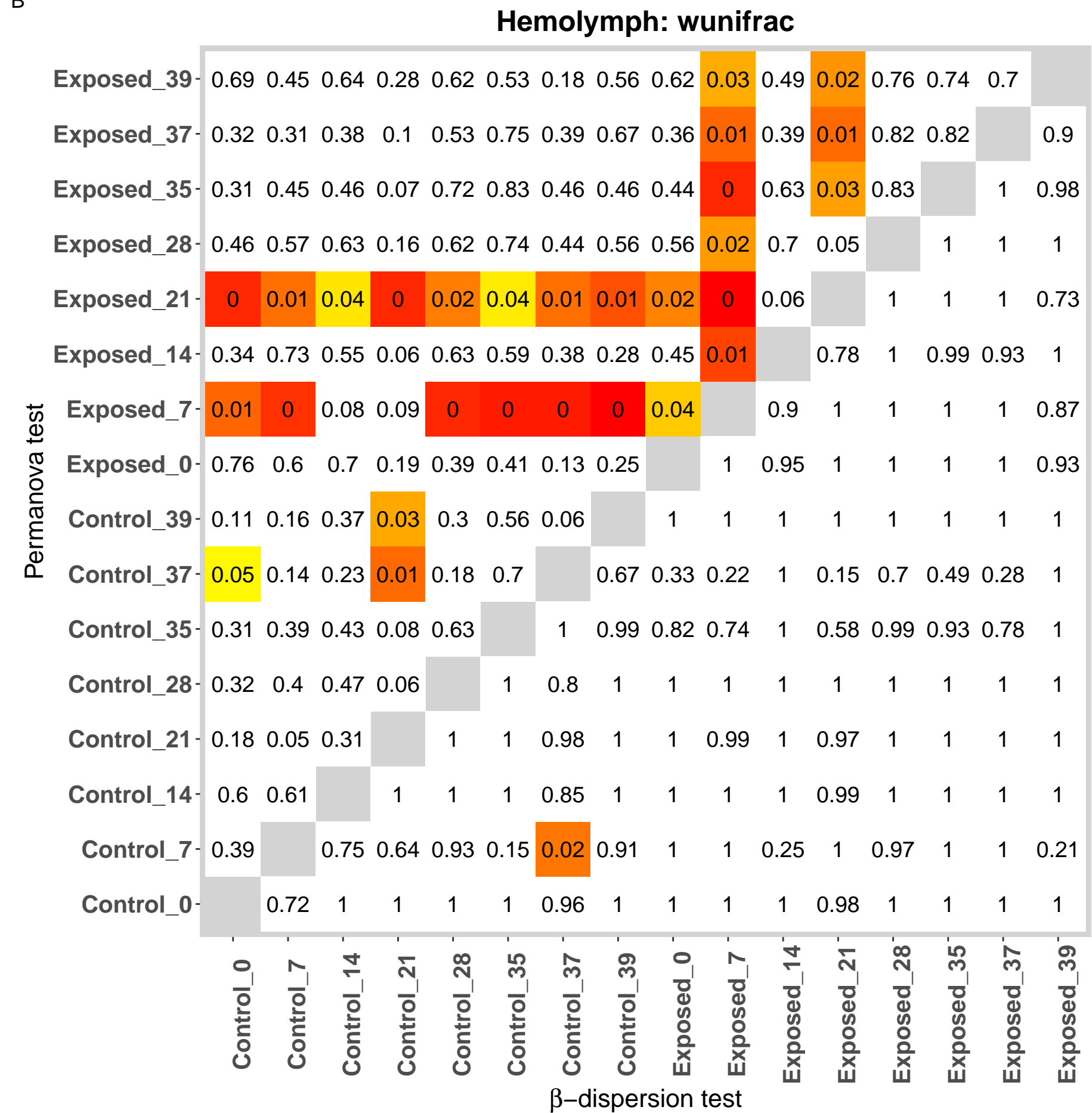

C

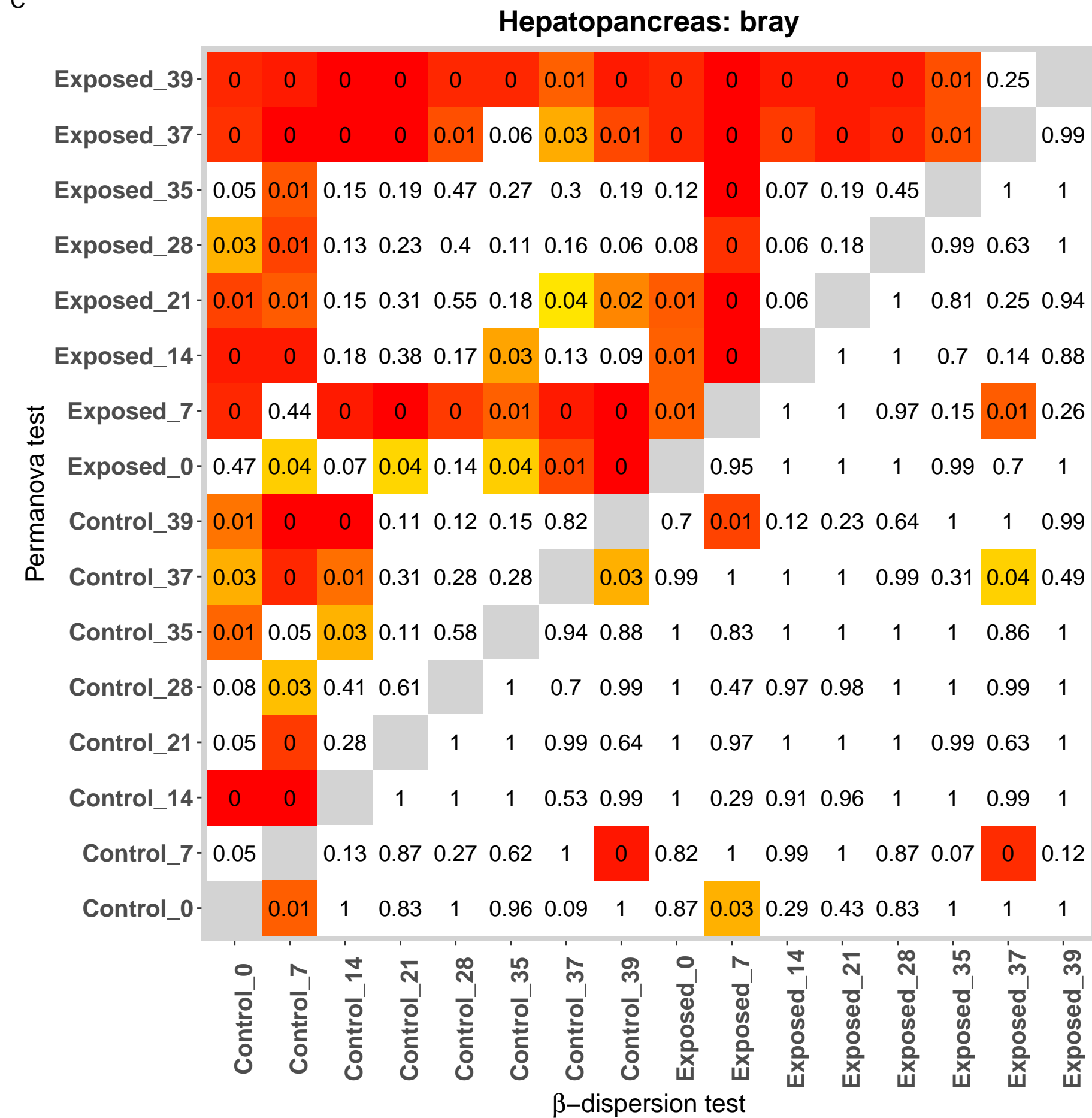

D

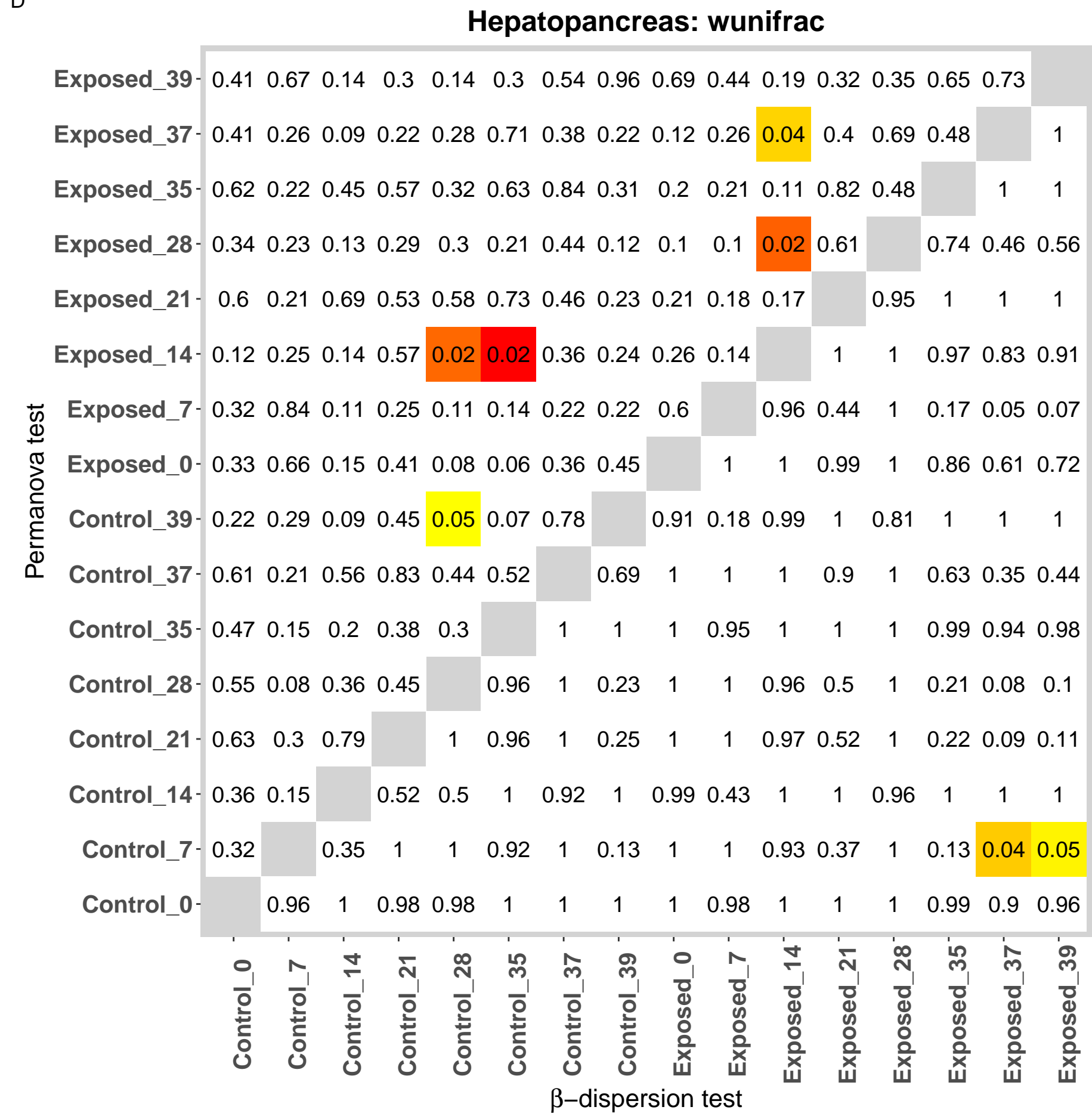

Supplement: Supplementary file 7 — Supplementary material 7: Figure 4. β-diversity: homogeneity of variances. [file 13071_2026_7299_MOESM7_ESM.pdf]

A

Hm: bray ~ Day

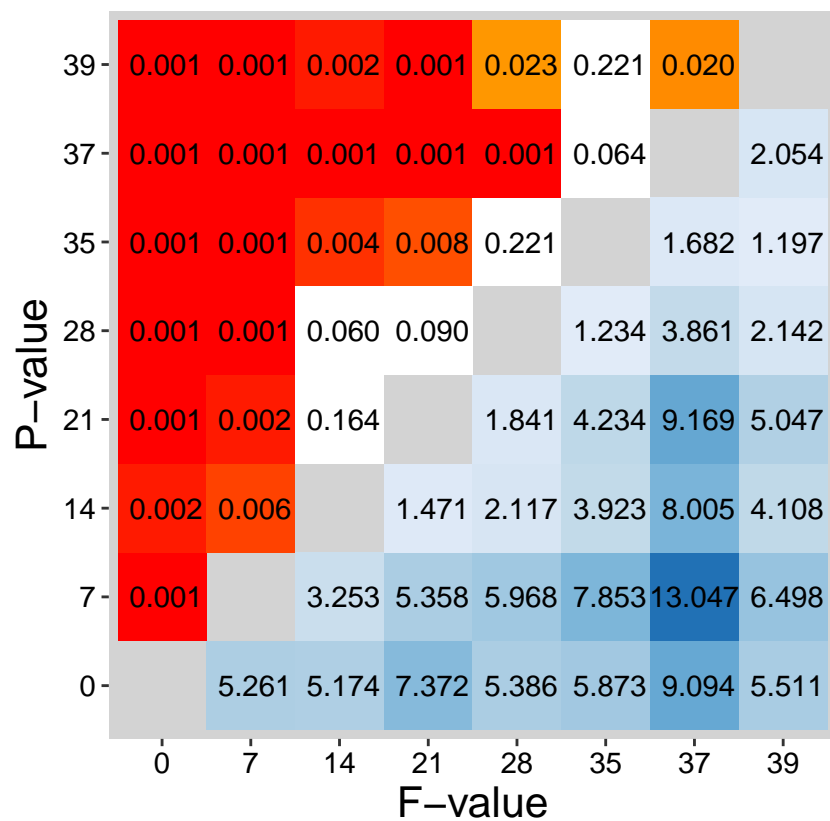

B

Hm: wunifrac ~ Day

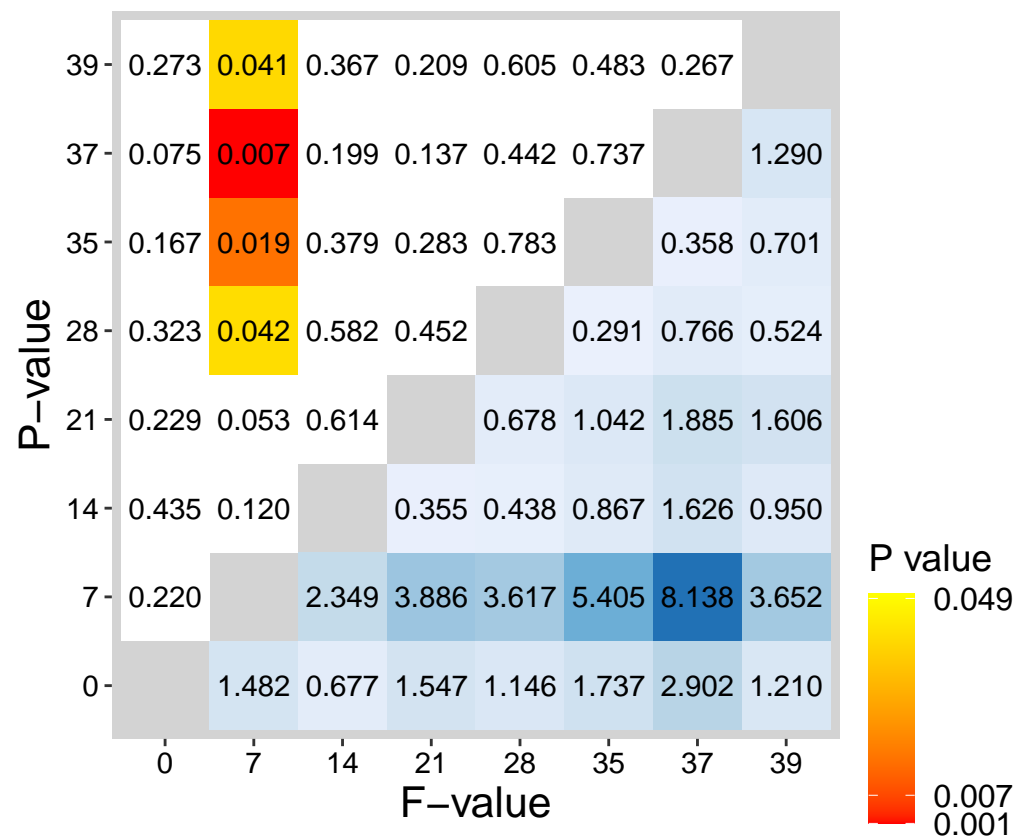

C

Hp: bray ~ Day

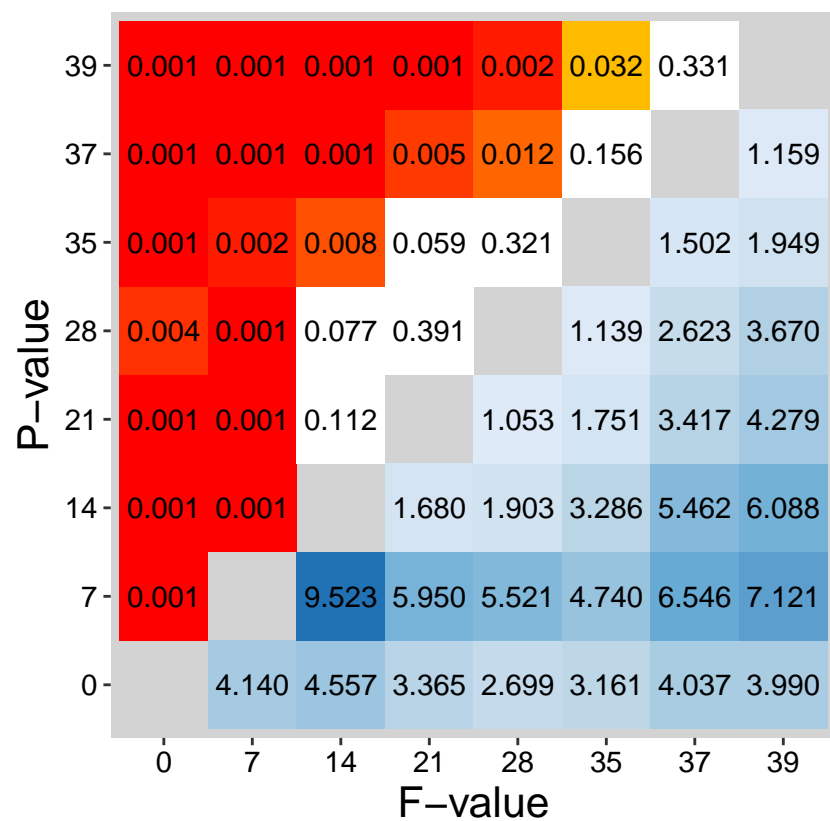

D

Hp: wunifrac ~ Day

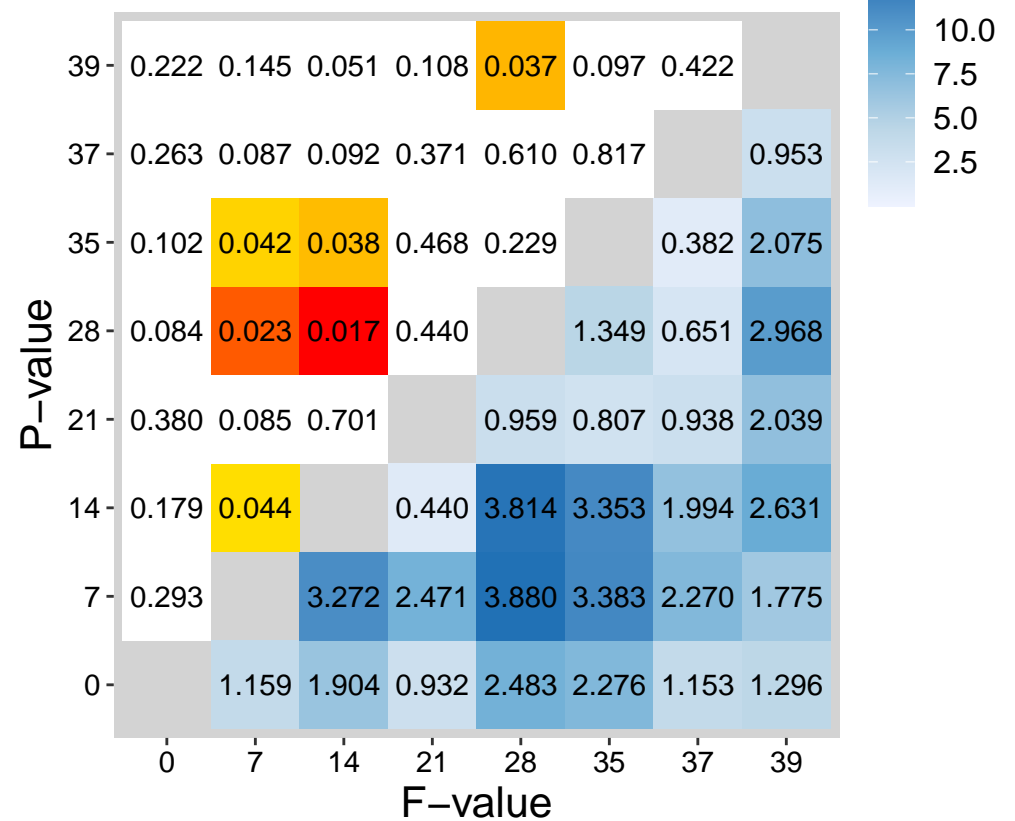

Supplement: Supplementary file 8 — Supplementary material 8: Figure 5. β-diversity: additive models day comparisons. [file 13071_2026_7299_MOESM8_ESM.pdf]

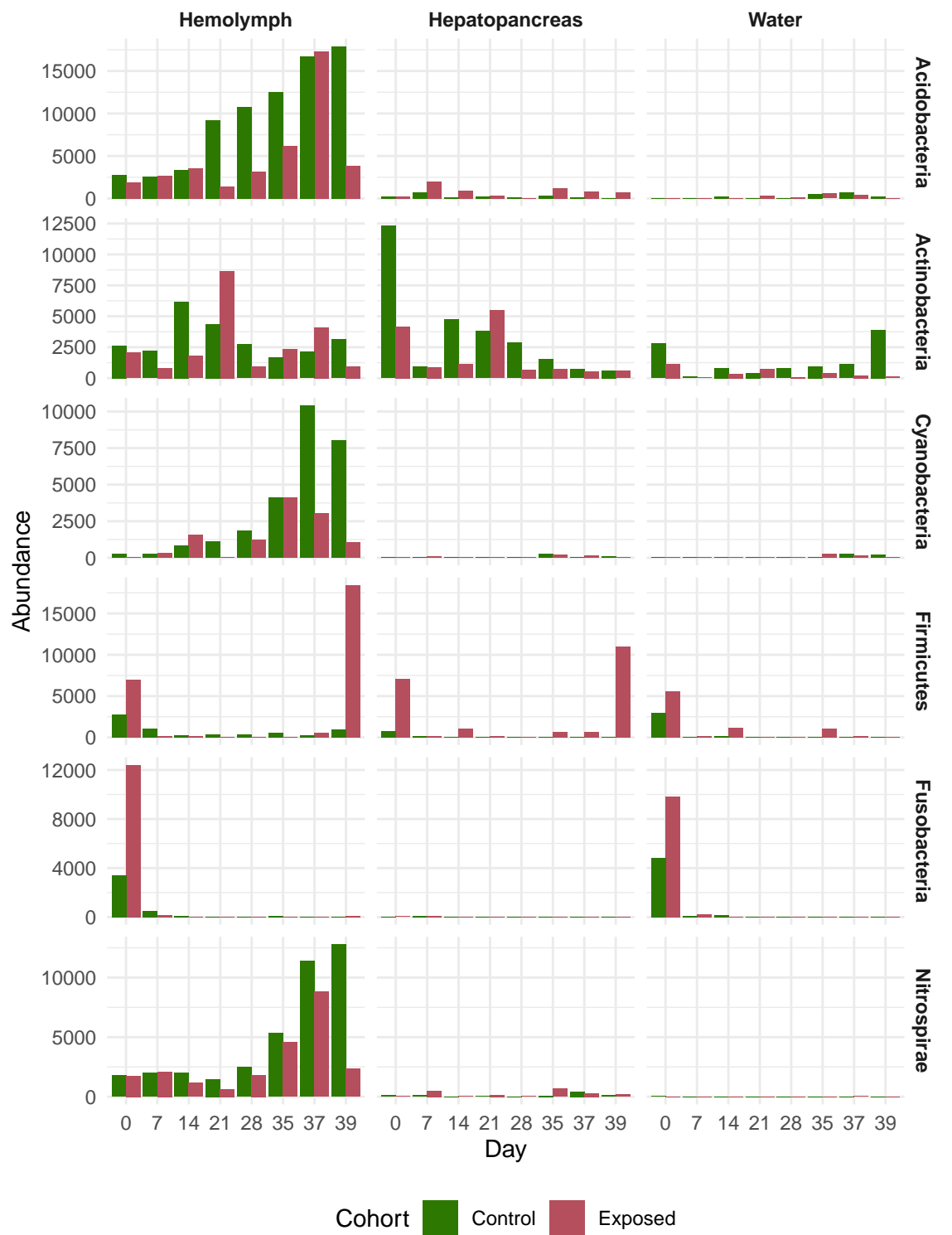

Supplement: Supplementary file 9 — Supplementary material 9: Figure 6. Abundance of the phyla showing the greatest variation during the course of infection. [file 13071_2026_7299_MOESM9_ESM.pdf]
